# Sex-dependent behavioral deficits and neuropathology in a maternal immune activation model of autism

**DOI:** 10.1038/s41398-019-0457-y

**Published:** 2019-03-28

**Authors:** Obelia Haida, Tareq Al Sagheer, Anais Balbous, Maureen Francheteau, Emmanuel Matas, Federico Soria, Pierre Olivier Fernagut, Mohamed Jaber

**Affiliations:** 1 Université de Poitiers, INSERM, Laboratoire de Neurosciences Expérimentales et Cliniques, Poitiers, France; 2 CHU Poitiers, Poitiers, France; 3 Université de Bordeaux, CNRS, Institut des Maladies Neurodégénératives, Bordeaux, France

## Abstract

Infections during gestation and the consequent maternal immune activation (MIA) increase the risk of developing neuropsychiatric disorders in infants and throughout life, including autism spectrum disorders (ASD). ASD is a neurodevelopmental disorder that affects three times more males than females and is mainly characterized by deficits in social communication and restricted interests. Consistent findings also indicate that ASD patients suffer from movement disorders, although these symptoms are not yet considered as diagnosis criteria. Here we used the double-stranded RNA analog polyinosinic:polycytidylic acid (poly I:C) MIA animal model of ASD in mice and explored its effects in males and females on social and motor behavior. We then investigated brain areas implicated in controlling and coordinating movements, namely the nigro-striatal pathway, motor cortex and cerebellum. We show that male mice are more affected by this treatment than females as they show reduced social interactions as well as motor development and coordination deficits. Reduced numbers of Purkinje cells in the cerebellum was found more widespread and within distinct lobules in males than in females. Moreover, a reduced number of neurons was found in the motor cortex of males only. These results suggest that females are better protected against developmental insults leading to ASD symptoms in mice. They also point to brain areas that may be targeted to better manage social and motor consequences of ASD.

## Introduction

Autism spectrum disorders (ASD) are a set of heterogeneous neurodevelopmental disorders characterized by persistent difficulties in verbal and nonverbal communication and restricted and repetitive patterns of behavior^1^. ASD can be diagnosed during childhood and affects 3 times more males than females^2^. In the absence of biological markers, ASD is currently diagnosed based on clinical scales and there is presently no cure but only symptomatic relief to some of its comorbidities such as anxiety, sleep disorders or seizures^3^.

Infection during pregnancy is known to alter neurodevelopment either following direct infection of the fetus or, more often, through the immune response of the mother. Maternal immune activation (MIA) is known to induce several neurological and psychiatric disorders, that, although symptomatically different, share some overlapping etiological and pathophysiological features^4^. These range from microcephaly, following the recently reported Zika virus infection for instance^5^, to schizophrenia and ASD. Indeed, bacterial, viral or parasite infections during pregnancy^6,7^ are reported to increase risks of ASD in offspring^8^. The infectious agents do not cross the placental barrier but it is rather the maternal cytokines and immune reaction that impact the fetal brain development following permeation of the placental compartment^9^. A study of over 10 000 cases of ASD in the Danish medical Register reported a clear link between viral infection during the first trimester of pregnancy and ASD; a weaker link was also established between bacterial infection during the second semester and ASD^10^.

MIA is modeled in animals mainly through injection of bacterial lipopolysaccharide or double stranded RNA analog polyinosinic:polycytidylic acid (poly I:C) that both have strong construct and face validity towards ASD and are preferred MIA paradigms compared to direct injection of bacteria or viruses^11,12^. Poly I:C injection to pregnant rodent females is considered to have a stronger construct validity than LPS^9^ and is generally performed at embryonic day 12.5 (E12.5), after the neural tube has closed and when progenitors are migrating, a time period when neurodevelopment is highly vulnerable to environmental insults. This treatment regimen has been shown to induce several cognitive an social features of ASD^9^ such as abnormal vocalization, deficits in social interaction and communication as well as altered cytokines levels within the cerebellum^13^. To this regard, a growing body of evidence is now pointing towards cerebellum abnormalities in ASD, where reduced numbers of Purkinje cells (PC) in post-mortem ASD brains has been documented^14,15^.

Here, we investigated the behavioral and cellular effects of a poly I:C injection to pregnant female mice at E12.5. We explored behavioral developmental milestones in males and females analyzed separately, followed by special focus on social interactions, gait and fine motor skills and coordination. We then determined the consequence of this treatment on cell integrity in several brain areas implicated in motor control and coordination, namely the nigro-striatal pathway, the motor cortex and the cerebellum. We demonstrate that a single injection of poly I:C to pregnant females induces not only long lasting abnormal social behavior in offspring but also developmental delays that were accompanied by reduced numbers of PC within the cerebellum and neurons in the motor cortex. Interestingly, these deficits were observed mainly in males, as females seemed less vulnerable to MIA both at the behavioral and cellular levels.

## Materials and methods

### Animals

Animal housing and experimental procedures were performed in accordance with the European Union directive (2010/63/EU) and validated by the regional ethical committee (Approval # 2015020415093780). C57BL/6J Mice (Charles River Laboratories, France) were housed in ventilated cages with access to food and water ad libitum. Room temperature was maintained at 23 °C on a 12 h light/dark cycle.

Pregnant mice received a single intraperitoneal injection of either poly I:C (20 mg/kg, Sigma, P1530, *n* = 22) or NaCl 0.9% (*n* = 11) at E12.5 as previously described^16^. Because poly I:C considerably increases the risk of resorption^17,18^, we used twice the number of mated females in the poly I:C group compared to control. Out of the 22 mated poly I:C females only 12 gave birth to pups (45.5% abortion) among which four litters died at neonatal age. All 11 saline treated females gave birth among which one litter died at neonatal age. Additionally, the mean number of pups per litter was significantly lower in poly I:C treated females compared to saline treated females (2.2 vs 6.7 pups per litter, Mann-Whitney: *p* < 0,001). At weaning (P21), pups from different litters were randomly allocated to four experimental groups depending on sex and prenatal treatment: saline males (*n* = 30), poly I:C males (*n* = 14), saline females (*n* = 30), poly I:C females (*n* = 10). The experiment timeline is presented in supplementary Figure 1. Behavioral tests were performed in the least stressing and challenging order to avoid potential training and learning effects as previously described with an ASD valproic acid (VPA) animal model^19^. Animals were tested during their light cycle and the investigators were blind to treatment assignment.

### Assessment of developmental milestones

During early postnatal life (1–3 weeks), pups were kept in their home cage and righting reflex and eye opening were assessed from P9 to P16. To assess righting reflex, mice were placed in the supine position and the time taken to right was monitored three times with a five-minute inter-trial interval. Eye opening was assessed daily at P12 to P16 and scored as either 0 = Both Eyes Closed, 1 = One Eye Open, or 2 = Both Eyes Open.

### SHIRPA primary screen

The SHIRPA primary screen^20^ was performed in a transparent Plexiglas arena (55 × 33 × 22 cm) with 11 × 11 cm square grid on the bottom, and a 3-mm metal wire crossing diagonally on top. The transfer reaction time was evaluated, followed by the wire maneuver test to assess motor coordination and muscle function. Negative geotaxis, used to assess postural stability and coordination in space, was evaluated as the time taken to turn and climb a 45° inclined grid^21^.

### Spontaneous activity in the cylinder

Spontaneous activity in the cylinder was performed at P30 as previously described^22^. Mice were put in a transparent Plexiglas cylinder, (diameter: 12 cm), and their activity was videotaped for 3 min. Number of rearing and time spent grooming were quantified.

### Motor coordination on the challenging beam

The challenging beam test was performed at P33 as previously described^23^. The beam consists of four Plexiglas sections (25 cm each) starting with a width of 3.5 cm and gradually narrowed to 0.5 by 1 cm decrements. Animals were first trained for 2 days to traverse the beam starting at the widest section and ending at the narrowest section that led into the home cage. On the test day, a mesh grid (1 cm squares) was placed over the beam surface. Animals were videotaped while traversing the grid- surfaced beam for five trials. Time to traverse, errors, number of steps and errors per step made by each animal were measured and averaged.

### Spatial, temporal, and kinetic parameters of gait

Gait was analyzed during spontaneous walk at P34 using an automated gait analysis system (Gaitlab, Viewpoint, France) as previously described^19^. The following parameters were analyzed: (i) stride length: distance between two consecutive placements of the same paw, (ii) limb base of support: distance between two pair prints at contact during each step cycle and (iii) pair gap: gap between the placement of the two trailing feet, which measures spatial coordination between the two pairs.

### Sociability in the three chambers test

Social interaction was assessed between P35 and P45 using the three-chambers test as previously described^19,24^. The first phase (PHASE-I) comprises two identical non-social stimuli (inverted wire-cups) placed in the opposite chambers. The second phase (PHASE-II) comprises a non-social stimulus and a social stimulus (a naïve mouse with no previous contact with the tested animal). Each phase was of 10 min during which time spent in each chamber was recorded. Subsequently, a sociability index (SI) was calculated as follows: (time exploring social chamber−time exploring non-social chamber)/(time exploring social chamber + time exploring non-social chamber).

### Tissue processing and immunohistochemistry

At the end of behavioral assays (P45), males and females from each group were randomly selected for histopathological analysis. Mice were deeply anesthetized with ketamine-xylazine (120–20 mg/kg) and transcardially perfused with 0.9% saline at 37 °C followed by 4% paraformaldehyde (PFA) at 4 °C. Brains were post-fixed in 4% PFA at 4 °C for 24 h before cryoprotection in 30% sucrose. Serial 50 µm (cerebellum) and 40 µm (striatum, cortex and substantia nigra) free-floating sections were collected and stored at −20 °C until use in an anti-freeze solution. PC within the cerebellum, dopaminergic neurons within the substantia nigra *pars compacta* (SNc) and neurons within the striatum and the motor cortex were quantified. Every fourth cerebellar section was mounted on gelatin-coated slides for PC quantification or microglia morphology analyses. PC were identified based on their morphology on cresyl violet staining as previously described^25^ and their phenotype was further confirmed using calbindin immunohistochemistry (1:2500; Swant, Cb-38a). Calbindin was not used for PC quantification as ASD inducing treatments can lead to reduced calbindin protein expression^26^. Microglia was revealed using a rabbit anti-Iba1 primary antibody (1:500; Wako, 019–19741) and a counterstaining with cresyl violet was used to identify granular and molecular layers. Every sixth section throughout the striatum, SNc and motor cortex was selected and processed for either neuronal nuclei antigen (NeuN) to quantify neurons in the striatum and the motor cortex, or for tyrosine hydroxylase (TH) immunoreactivity to quantify dopaminergic neurons in the SNc. Sections were incubated for 1h30 in a blocking solution (3% bovine serum albumin, 0.3% Triton X-100 in PBS 1 M, pH 7.4). Rabbit anti-NeuN (1:500; abcam, Ab177487) or mouse anti-TH (1:5000; Immunostar, 22941) antibodies were applied overnight at 4 °C. Biotinylated anti-rabbit or anti-mouse IgG was used as secondary antibody (1:250; Vector laboratories, BA-1000 and BA-9200) for 1 h at room temperature. Signal was amplified with an ABC Elite kit and revealed with diaminobenzidine (Vector Laboratories). Sections were mounted on gelatin-coated slides and processed for cresyl violet counterstaining.

### Stereology

Stereological estimates were performed using the optical fractionator method and systematic random sampling to obtain the total number of cerebellar PC, motor cortex neurons, striatal neurons and dopaminergic nigral neurons. Each region of interest was outlined based on the Franklin and Paxinos’s mouse brain atlas^27^ at ×2.5 objective and neurons were counted at ×40 objective using Mercator image analysis system (Explora Nova, France). Upper and lower guard zones of 1 µm were set at the top and bottom of the section to exclude lost profiles and each neuron or visible nucleus was counted (see supplementary Table 1 for stereology parameters).

### Microglial morphology

Z-stack images of Iba1-stained cerebellar sections were taken at ×40 objective in different parts of the sublobule Crus II in males, and distinguishing between the granular and molecular layers. Images were then processed for segmentation and fractal analysis by a semi-automated method using ImageJ software as previously described^28^. Briefly, diaminobenzydine and cresyl violet stainings were artificially separated by color deconvolution. Resulting z-stacks were converted to maximal projection images with the stack ‘sum’ function before thresholded binarization. Area and perimeter of cells and their convex hulls were measured with ImageJ default tools to calculate shape descriptors. Fractal box counting dimension, form factor (4π x cell area/cell perimeter²), convexity (convex hull perimeter/cell perimeter) and solidity (cell area/convex hull area) were calculated for at least 20 cells per animal.

### Statistical analyses

Data are expressed as mean ± Standard Error of the Mean and analyzed using GraphPad Prism-7 software (La Jolla, California, USA). Normality and equality of group variances were tested by Shapiro-Wilk and Brown-Forsythe tests, respectively. Data having a Gaussian distribution and equal variance were analyzed using two-way analysis of variance (ANOVAs) followed by Fisher’s LSD post-hoc multiple comparisons test, except when comparing only two groups where a Student’s *T*-test was performed. Kruskal-Wallis test followed by Dunn’s post-hoc test or Mann–Whitney test was applied when samples did not follow a normal distribution or had unequal variance. For all analyses, a *p* value < 0.05 was considered significant.

## Results

### Post-natal development delays in poly I:C mice

**Fig. 1:**
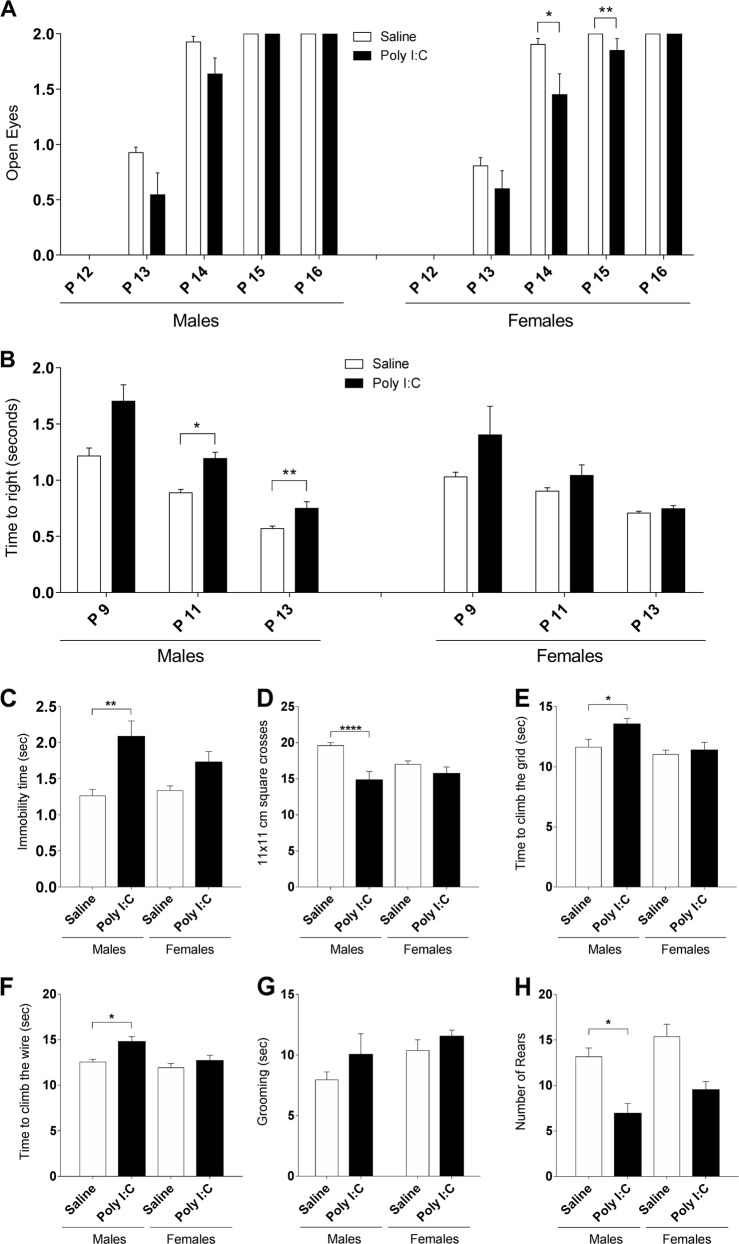
Prenatal exposure to poly I:C leads to developmental delays in males and females and altered spontaneous activity and motor coordination in males. **a** Delay in eye opening was measured as the mean number of open eyes (0, 1, or 2) per group from P12 to P16 in males (left) and females (right). **b** Increased latency to right at P11 and P13 in poly I:C males. **c** Immobility time when transferred to the SHIRPA arena is increased only in poly I:C males. **d** Locomotor activity after the transfer to the SHIRPA arena. Only poly I:C males showed a decrease in this parameter. **e** Time to climb on the grid was found increased in poly I:C mice compared to saline in males only. **f** Significant increase in time spent climbing the wire was found only in poly I:C males. **g** No significant change in the time spent grooming was found in poly I:C mice compared to saline. **h** Poly I:C prenatal exposure significantly decreased the number of vertical rears in the cylinder in poly I:C males only. Poly I:C males *n* = 11; saline males *n* = 27; poly I:C females *n* = 10; saline females *n* = 30. Data expressed as mean ± SEM; two-way ANOVA followed by Fisher’s LSD post-hoc (**d**, **e**, and **g**), and Kruskal-Wallis test followed by Dunn’s multiple comparisons test (**a**, **b**, **c**, **f**, and **h**) (*p < 0.05; **p < 0.01; ****p < 0.0001)

To determine the effect of prenatal exposure to poly I:C on general postnatal development we investigated eye opening delays and righting reflex at P9-P16. In the eye opening measurements (Fig. 1a), Kruskal-Wallis test indicated a significant group effect at P13 (*p* < 0.05), P14 (*p* < 0.01) and P15 (*p* < 0.01). A significant delay of eye opening was observed in poly I:C females at P14 and P15 (Dunn’s post-hoc test, *p* < 0.05 and *p* < 0.01, respectively). For the righting reflex, Kruskal-Wallis test revealed a significant group effect at P9 (*p* < 0.01), P11 (*p* < 0.05) and P13 (*p* < 0.001). A significant increase of the time to right was observed in poly I:C males at P11 and P13 (Dunn’s post-hoc test, *p* < 0.05 and *p* < 0.01, respectively). These results indicate that both males and females exposed prenatally to poly I:C manifest developmental delays, although different in nature.

### Poly I:C males show altered spontaneous activity and motor coordination

At P30, poly I:C mice showed altered behavior in different SHIRPA primary screen and cylinder test parameters (Fig. 1c–h). Time spent immobile following transfer to the SHIRPA arena was assessed. Kruskal-Wallis test revealed a significant group effect (*p* < 0.001) with poly I:C males showing ~65% increase in immobility compared with saline (Dunn’s multiple comparison test, *p* < 0.01, Fig. 1c). Locomotor activity assessment (Fig. 1d) showed an effect of treatment [*F* (1,74) = 18.72, *p* < 0.0001], and a treatment x sex interaction [*F* (1,74) = 6.124, *p* < 0.05]. Fisher’s LSD post- hoc analysis showed a significant (−24%) decrease in locomotor activity only in poly I:C males (*p* < 0.0001). We then assessed postural stability and coordination in space using negative geotaxis measured as the time needed to climb a 45° inclined grid (Fig. 1e). We found an effect of sex [*F* (1,74) = 4.486, *p* < 0.05] and Fisher’s LSD post-hoc test indicated a significant increase of the time needed to climb the grid in poly I:C males (*p* < 0.05) but not in females. Finally, we implemented the wire maneuver test to assess motor coordination and muscle function (Fig. 1f). Kruskal-Wallis test showed a significant effect of group (*p* < 0.01) and Dunn’s multiple comparison test determined that only poly I:C males were statistically different from saline and spent more time to climb the metal wire (+17%, *p* < 0.05).

Repetitive and restricted behaviors are among the core symptoms and the earliest signs of ASD^1^. We assessed repetitive grooming and rearing behaviors in a new environment using the Plexiglas cylinder test for 3 minutes. Kruskal-Wallis test indicated an effect of group in the time spent grooming (*p* < 0.05, Fig. 1g). However, Dunn’s multiple comparison test indicated no significant change in both poly I:C males and females compared to their saline counterparts. Poly I:C treatment had a significant effect on rearing behavior (Kruskal-Wallis, *p* < 0.01) with a significant 50% decrease of rearing in males (Dunn’s multiple comparison test *p* < 0.05, Fig. 1h) while a trend was observed in females (*p* = 0.1014). These results indicate that prenatal exposure to poly I:C leads to exploratory and motor coordination deficits mainly in males.

### Poly I:C treatment did not affect gait nor walking skills

**Fig. 2:**
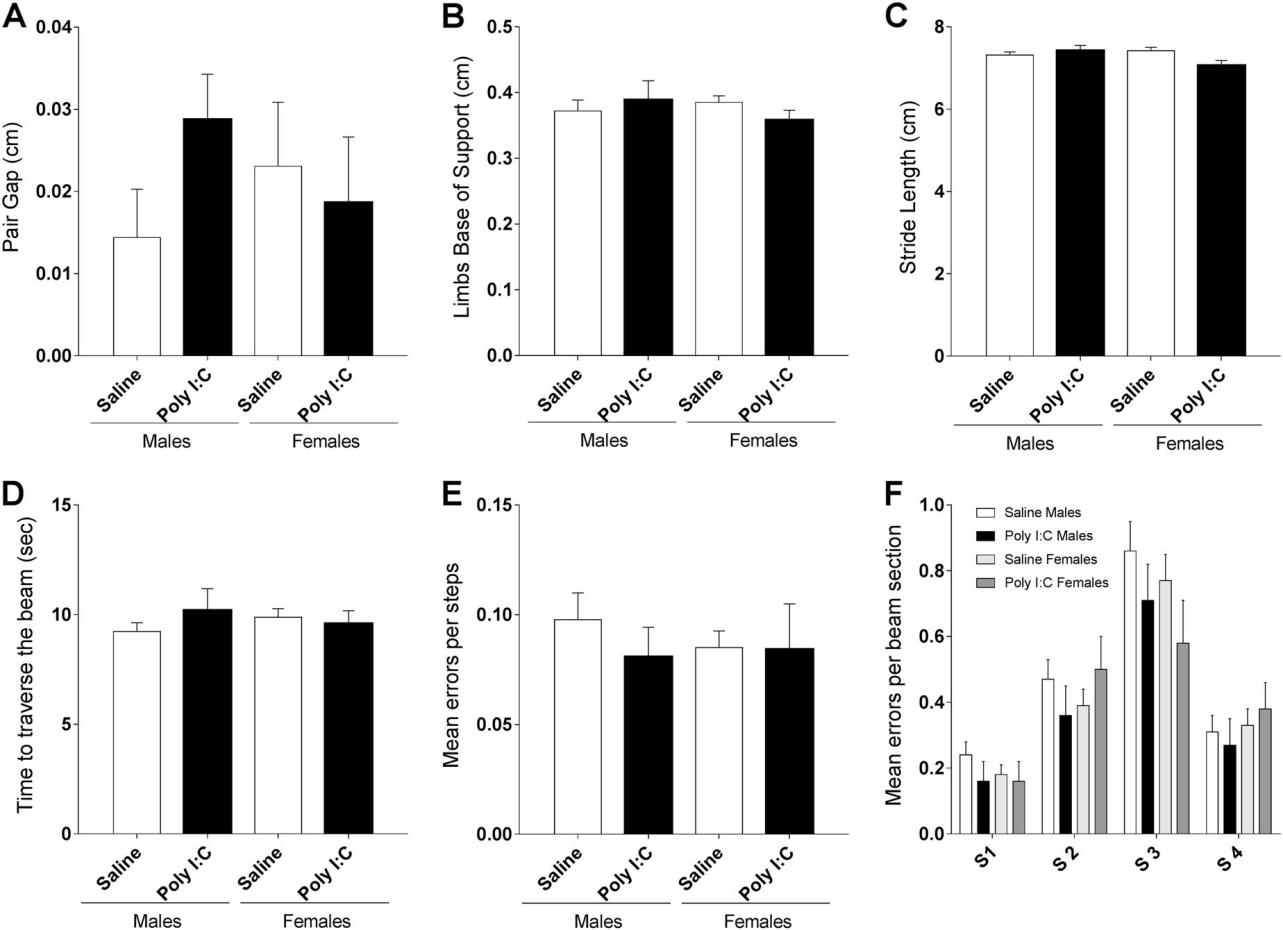
Prenatal exposure to poly I:C does not affect gait. Poly I:C mice show no significant change in pair gap (**a**), limbs base of support (**b**) or stride length (**c**). **d** Poly I:C treatment did not affect the time needed to traverse the beam nor the number of errors per steps crossing the beam (**e**). Poly I:C prenatally exposed males (black bars) and females (dark gray bars) showed no change in the number of errors per steps on different beam sections, in comparison to saline (**f**). Saline males *n* = 27; Poly I:C males *n* = 11; Saline females *n* = 30; Poly I:C females *n* = 10. Data expressed as mean ± SEM; two-way ANOVA followed by Fisher’s LSD post-hoc (**b**, **c**, **d**, and **f**), and Kruskal-Wallis test followed by Dunn’s multiple comparisons test (**a**, **e**)

We analyzed gait at P33–34 as several studies in ASD patients clearly pointed to gait disturbances, including reduced stride length and increased base of support^29,30^. Poly I:C treatment did not affect paw area [*F* (1,74) = 0.03356, *p* = 0.8551], nor speed [*F* (1,74) = 3.451, *p* = 0.0672] or regularity of the run [*F* (1,74) = 5.328e-005, *p* = 0.9942], neither in male or female mice. Kruskal-Wallis test indicated no effect of treatment or sex on the pair gap of walking (Fig. 2a). Additionally, two-way ANOVA revealed no significant effect of group [*F* (3,148) = 0.7706, *p* = 0.5122] on limbs base of support (Fig. 2b) or stride length [*F* (1, 74) = 0.1638, *p* = 0.6868] (Fig. 2c). Two-way ANOVA showed an effect of sex [*F* (1, 74) = 4.801, *p* < 0.05] on stride length but with no significant change in both poly I:C males and females in comparison to saline controls (Fisher’s LSD post-hoc analysis). On the challenging beam test, statistical tests indicate no effect of treatment or sex on the time needed to traverse the beam sections (Fig. 2d). In addition, the number of errors per step (Fig. 2e) and the number of errors at each beam section (Fig. 2f) were not affected by the treatment. These results indicate that poly I:C prenatal exposure did not induce gait disturbances in mice.

### Reduced sociability in poly I:C males

**Fig. 3:**
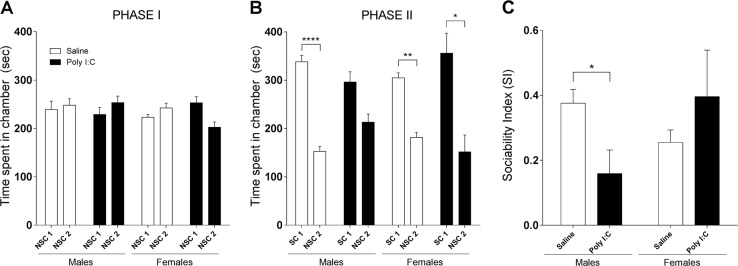
Prenatal exposure to poly I:C leads to reduced social behavior in males. Sociability was assessed through the three-chambers test. **a** PHASE I: time spent in nonsocial chamber-1 (NSC1) *versus* nonsocial chamber-2 (NSC2) is unaffected by treatment or sex. **b** PHASE II: time spent in the social chamber-1 (SC1) is significantly higher than time spent in NSC2 for each groups except for poly I:C males. In line with this finding, sociability index (SI) indicates that poly I:C prenatally treated males have a significantly decreased sociability (**c**). Saline males *n* = 10; Poly I:C males *n* = 8; Saline females *n* = 15; Poly I:C females *n* = 5. Data expressed as mean ± SEM; Kruskal-Wallis test followed by Dunn’s multiple comparisons test (**a** and **b**), and two-way ANOVA followed by Fisher’s LSD post-hoc (**c**) (*p < 0.05; **p < 0.01; ****p < 0.0001)

One of the hallmarks of ASD are deficits in social interactions^1^. Social behavior of mice prenatally exposed to poly I:C was assessed at age of 5–6 weeks (P35-45) using the three-chambers test (Fig. 3) as previously described^19^. None of the treatment groups, regardless of sex, showed a preference to any of the chambers during the 10 min habituation (PHASE I) (Fig. 3a). In Phase II, Kruskal-Wallis test detected a significant effect of group (*p* < 0.0001). Indeed, while saline males spent more time in the social chamber than in the non-social one (69% versus 31%, *p* < 0.001), poly I:C males spent 58% of their time in the social chamber versus 42% in the non-social one (*p* > 0.999) showing no significant preference towards social interaction (Fig. 3b). Dunn’s multiple comparison test showed that both saline and poly I:C females spent more time in the social chamber (*p* < 0.01 and *p* < 0.05, respectively). These findings were further confirmed when expressed as SI as two-way ANOVA showed a sex × treatment interaction effect [*F* (1, 34 = 7.502, *p* < 0.01) with a significant 57% decrease in sociability in poly I:C males (Fisher’s LSD, *p* < 0.05) while females had similar SI regardless of the treatment (Fig. 3c). These results reveal that poly I:C prenatal exposure impairs sociability in males, but not in females. This is in accordance with the sex differences of ASD in clinical settings where ASD is 3 times more diagnosed in males than in females^2^.

### Reduced number of neurons in poly I:C mice

**Fig. 4:**
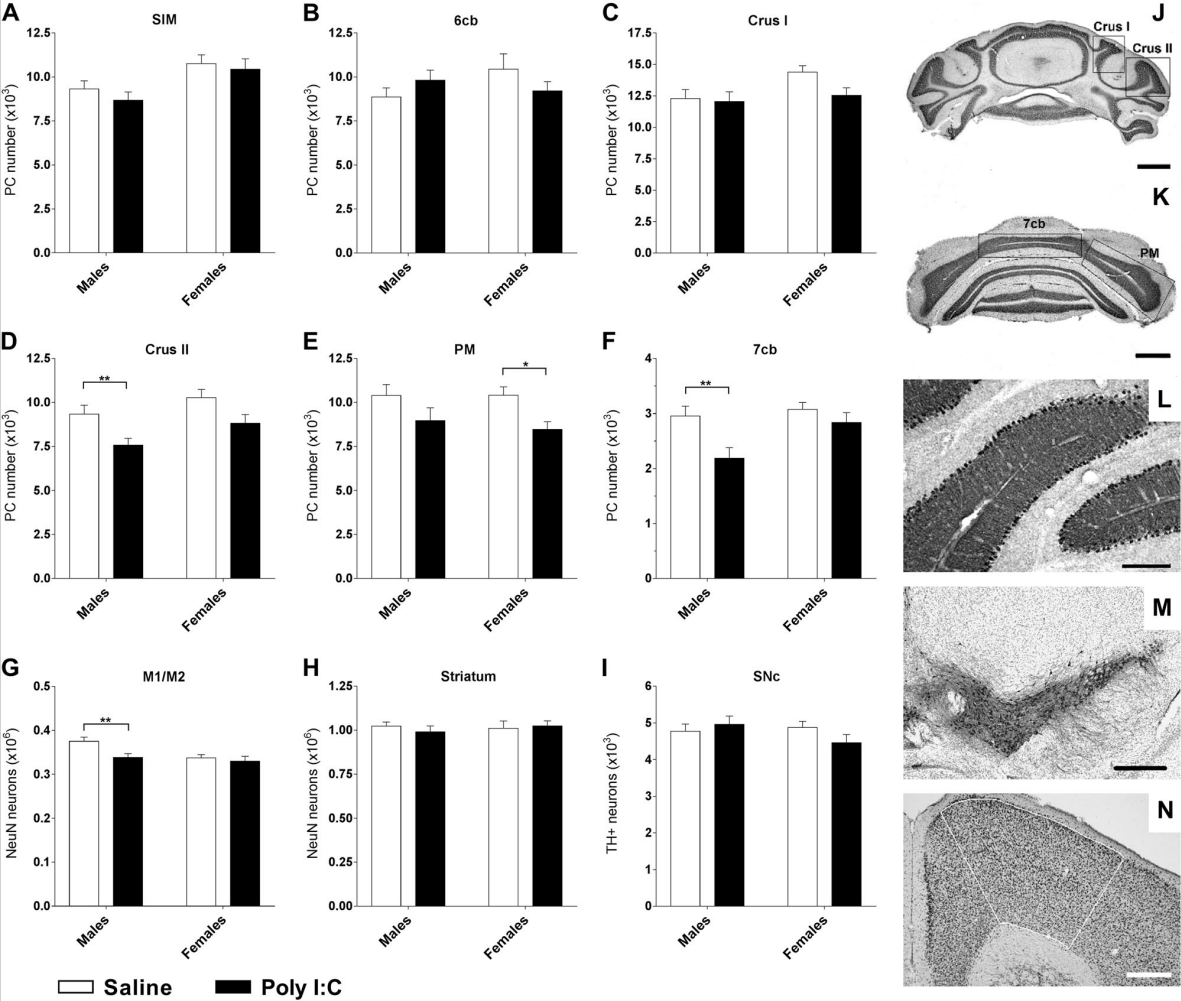
Prenatal exposure to poly I:C leads to reduced numbers of neurons in the cerebellum and motor cortex in a sex-dependent manner. **a**–**f** Stereological Purkinje cells (PC) counts on coronal sections of the cerebellum. **j**, **k** Photomicrographs of the different sub-lobules of lobule VII, scale bars = 1 mm. **l** Illustration of the monolayer organization of PC in the cerebellar cortex after DAB-calbindin immunolabeling, scale bars = 200 µm. No decrease of the PC number occurred within lobule VI, neither in the hemispheric part, SIM (**a**) nor in the vermal part, 6cb (**b**). In the hemispheric part of the lobule VII, the number of PC in Crus I (**c**) was not affected by treatment whereas a significant reduction was found in Crus II in poly I:C males (**d**) and in paramedian lobule (PM) in females (**e**). In the vermal part corresponding to the sub-lobule 7cb (**f**), the number of PC was significantly decreased in poly I:C males. **g** Decreased number of NeuN-stained neurons in M1/M2 motor cortex in poly I:C males (outlined area on (**n**), scale bars = 400 µm). **h** Unaffected numbers of NeuN-stained striatal neurons or (**i**) tyrosine hydroxylase-positive neurons in the Substantia Nigra *pars compacta* (SNc), (**m**) DAB-TH immunolabelling, scale bars = 400 µm). *n* = saline males/poly I:C males/saline females/poly I:C females; *n* (**a**) = 11/10/10/9; *n* (**b**) = 14/11/10/8; *n* (**c**) = 13/11/9/9; *n* (**d**) = 13/10/9/8; *n* (**e**) = 12/8/9/8; *n* (**f**) = 12/10/8/8; *n* (**g**) = 12/9/10/9; *n* (**h**) = 12/9/10/9; *n* (**i**) = 12/9/11/9. Data expressed as mean ± SEM; two-way ANOVA followed by Fisher’s LSD or Kruskal-Wallis test followed by Dunn’s multiple comparisons test (**b**) (*p < 0.05; **p < 0.01)

Given the behavioral motor deficits observed, we aimed at determining the effect of poly I:C treatment on cell numbers in major brain areas involved in motor control such as the cerebellum, the nigro-striatal pathway and the motor cortex. In the cerebellum, we focused our analyses on the two lobules that have been demonstrated to be the most affected in ASD: lobule VI and VII^31,32^. In lobule VI, we did not observe any effect of treatment on PC number (Fig. 4a, b). However, two- way ANOVA indicated an effect of sex in the sublobule SIM [*F* (1, 36) = 10.08, *p* < 0.05]. In the sublobule 6cb, Kruskal-Wallis test showed no effect of group. In lobule VII, we observed an effect depending on the sub-lobules investigated (Crus I, Crus II, PM and 7cb, Fig. 4c–f, j–l) and on the sex of poly I:C mice. In Crus II, two-way ANOVA revealed a significant effect of treatment [*F* (1, 36) = 10.65, *p* < 0.01] and sex [*F* (1, 36) = 4.923, *p* < 0.05] but no sex × treatment interaction [*F* (1, 36) = 0.09464, *p* = 0.7601]. Fisher’s LSD post-hoc test revealed a significant reduction of the number of PC (~15%) in poly I:C males in the sub-lobule Crus II (*p* < 0.01, Fig. 4d) but no significant difference in females. There was also a significant effect of treatment in 7cb [*F* (1, 34) = 7.65, *p* < 0.01] and a significant effect of sex [*F* (1, 34) = 4.529, *p* < 0.05] but no sex x treatment interaction [*F* (1, 34) = 2.182, *p* = 0.1489]. A significant reduction of the number of PC (~26%) was found in poly I:C males (Fisher’s LSD test: *p* < 0.05) but not in females (Fig. 4f). Two-way ANOVA showed a treatment effect [*F* (1, 33) = 7.74, (*p* < 0.01)] in the paramedian (PM) lobule (Fig. 4e) where PC number was significantly reduced by ~19% in females poly I:C, but not in males, as revealed by Fisher’s LSD test. These findings indicate that poly I:C treatment induced a sex-dependent decrease of PC number within sex-specific regions of the cerebellum.

We also quantified NeuN immunoreactive neurons in the primary and the secondary motor cortex (Fig. 4n). Two-way ANOVA analysis revealed a significant effect of treatment [*F* (1, 37) = 5.706, *p* < 0.05] and sex [*F* (1, 37) = 6.462, *p* < 0.05] but no sex × treatment interaction [*F* (1,37) = 2.625, *p* = 0.1137]. Post-hoc tests showed a significant decrease by 10% of cortical neurons in poly I:C males (*p* < 0.01) but not in females (*p* = 0.5953) (Fig. 4g). We also investigated whether the nigro-striatal pathway could be implicated in the motor deficits observed. Our results showed that neither the number of striatal neurons (Fig. 4h), nor the number of neurons in the SNc were altered (Fig. 4i, m), indicating that motor or exploratory deficits following poly I:C treatment are not associated with reduced numbers of striatal or nigral neurons. Indeed, two-way ANOVA did not reveal any effect of treatment or sex neither in the SNc [*F* (1, 36) = 0.3004, *p* = 0.587 and *F* (1, 36) = 0.9489, *p* = 0.3365, respectively], nor in the striatum [*F* (1,36) = 0.08735, *p* = 0.7693 and *F* (1,36) = 0.1277, *p* = 0.723, respectively]. These results indicate that prenatal exposure to poly I:C lead to reduced number of PC in cerebellar lobules, such effect being more widespread and distinct in males than in females.

### No changes in the microglia morphology at P45 in Crus II

**Fig. 5:**
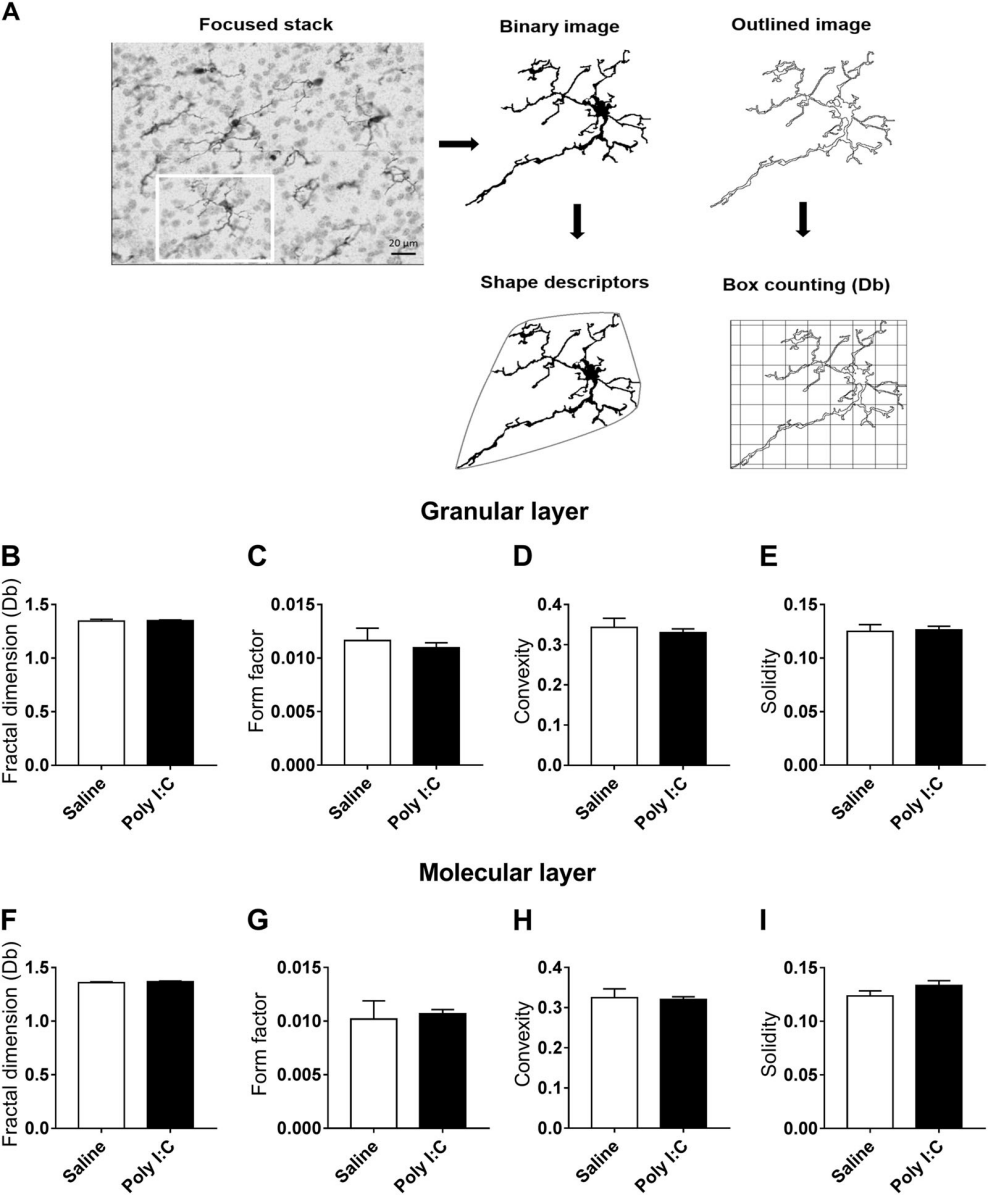
Poly I:C prenatal treatment does not induce alterations in the microglial cell morphology. **a** Workflow for the morphological analysis of individual Iba1 + microglial cells from DAB-staining images. Binary images were used to calculate shape descriptors and outlined images to quantify box counting fractal dimension. Quantification of fractal dimension and morphology descriptors in microglia from the sublobule Crus II of saline and poly I:C male mice in the granular layer (**b**–**e**) and in the molecular layer (**f**–**i**). Saline males *n* = 6; Poly I:C males *n* = 6. Data are presented as mean ± SEM; Data were analyzed using student’s *T*-test (**b**, **f**) or Mann-Whitney test (**c**, **d**, **e**, **g**, **h**, **i**) when sample did not follow a Gaussian distribution

Following the observation of a reduced number of PC in Crus II in poly I:C males, we then investigated whether MIA could have induced long-lasting microglial activation in this area (Fig. 5). The majority of the cells had a small soma with multiple processes, features that characterize quiescent microglial cells (Fig. 5a). Fractal analysis showed no change of Iba1 positive microglial cells morphology between the saline and the poly I:C male mice, neither in the granular layer (Fig. 5b–e), nor in the molecular layer (Fig. 5f–i). Poly I:C treatment did not affect any parameters of microglia morphology investigated, neither in the granular layer nor in the molecular layer: fractal dimension (*T*-test: *p* = 0.9247; *p* = 0.4415, respectively), form factor (Mann-Whitney: *p* = 0.9372; *p* = 0.1320, respectively), convexity (Mann-Whitney, *p* = 0.9372; *p* = 0.6991, respectively) and solidity (Mann-Whitney, *p* = 0.4848; *p* = 0.1797, respectively). These results indicate that there is no protracted microglial activation in Crus II in poly I:C males.

## Discussion

This study aimed at investigating several behavioral and cellular parameters related to ASD in male and female mice that were exposed to a MIA procedure during their prenatal life. For this, we explored social interactions, motor and gait performances at different time points and determined their histological correlates in brain areas implicated in motor control, regulation and coordination. Our results indicate that while poly I:C prenatal exposure leads to delayed development in both male and female pups, only periadolescent males, but not females, showed increased immobility in a novel environment, decreased motor/exploratory activity and motor coordination deficits as attested by increased time necessary to climb a grid or a wire. Social behavior in poly I:C young adult male was also significantly reduced while poly I:C female sociability did not differ from saline. However, neither poly I:C males nor females showed deficiencies in fine motor skills evaluated using the narrowing beam walking paradigm nor in gait. Interestingly, behavioral deficiencies observed mainly in poly I:C males were mirrored by restricted cell number reduction in the lateral parts of the cerebellum, its vermis, and in the motor cortex. Poly I:C females showed a reduced PC number only in the PM lobule of the cerebellum, and that was different from the affected lobules in poly I:C males. In line with these findings, we also recently reported similar results using the VPA ASD animal model where males were more affected by the prenatal treatment than females whether at the behavioral or cellular levels^19^.

Most previous studies using the poly I:C prenatal exposure rodent model have focused only on males^33^ or have pooled together males and females^16^ rendering it difficult to determine potential sex differences following MIA. This is of relevance given that ASD ratio is 3:1 in males^2^ and being a male constitutes a major risk factor in developing neurological or psychiatric disorders^34^. Poly I:C-induced MIA is a common procedure used to generate animal models of ASD, but also depression and schizophrenia^35–37^. These psychiatric pathologies, although different in their expression, seem to share to some extent overlapping clinical and therapeutic features and common biological mechanisms such as epigenetic modifications involving histone acetylation and promoter methylation^38^. The link between MIA and ASD is potent and has been confirmed in human^10,39^ and animal models^33,35,40^. The poly I:C animal model of ASD has been repeatedly validated and is recognized to provide strong construct and face validity^16,35,41^. While this manuscript was in the submission process a review article proposing guidelines for the use of MIA models was published and that aims to limit discrepancies in the MIA procedures that may account for variations between findings^42^. The poly I:C procedure used here has been widely documented and consistently showed induction of MIA (as evidenced for example by elevation of cytokine blood levels in treated pregnant females) and behavioral consequences in offspring^43,44^.

Previous reports have shown that poly I:C prenatal treatment induces decreased social interaction with normal grooming behavior^35^ and decreased motor performance in a rotarod task only in males^33^. Additionally, prior research using similar dose and time of exposure of poly I:C have found that this treatment induces in the offspring males decreased exploration in open-field as well as reduced preference for the social chamber in three chambers test^41^. However, and to the best of our knowledge, this is the first study exploring in detail motor behavior, fine motor skills and gait in both sexes in the poly I:C mouse model. This is also the first study that focuses on cerebellar sub-regions, motor cortex and the nigrostriatal pathway in these animal models and reporting restricted reduction of neurons in distinct regions of the cerebellum and the motor cortex and that were mainly observed in poly I:C males.

The finding that gestational MIA can induce ASD-like postnatal and durable behavior abnormalities underlines the roles played by the prenatal environment in shaping brain development and in provoking lasting complex behaviors. Alterations in neuronal development occurring around E12 in rodents can result in severe abnormalities as this is a key period of neuronal proliferation, migration, differentiation, synaptogenesis, apoptosis and myelination^45^.

Cerebellar lobules VI and VII play a major role in movement regulation, exploratory behavior, stereotyped and repetitive behaviors, and oculomotor activity^46–48^ but also in cognitive functions such as speech^49^. PC are the integrating center of the cerebellum and the sole output from the cerebellar cortex. Several studies have shown a decrease number of theses neurons in ASD^14,15,50,51,55^ and in ASD models^16,19,33^. In this study, we have demonstrated a reduction of the PC number in the cerebellum of mice prenatally exposed to poly I:C and that were different in their extend and regional specificity in males and females. Most previous studies with animal modes of ASD used either only males^33^ or combined males and females within the same group^16^. In this latter reference, the authors showed a 26% reduced number of PC in the vermal lobule VII, but not in lobule VI. This finding was later confirmed by Naviaux et al. in 2013 who reported a decrease of the PC number by 63% at the age of 16 weeks^33^. Our results are thus in accordance with these studies indicating no change in the number of PC in the lobule VI and a significant decrease in the lobule VII, of 15 to 26% magnitude at the age of 45 days. Moreover, we extend these findings and show here that this reduction appears to be restricted to the sub-lobules Crus II and 7cb in males and in PM in females, cerebellar regions that are easier to delineate with coronal sections as used here compared to sagittal sections used in prior studies. In human, Crus II plays an important role in speech, social cognition, stereotyped and repetitive behaviors, movement regulation and oculomotor activity^49,52,53^ all reported to be affected in ASD^54^. The PC of this region are inter-connected with the area 46 of the dorsolateral prefrontal cortex, a region involved in working memory, attention, movement regulation and organization. Deficits in PC in this cerebellar area could underlie some ASD symptoms such as language/communication disturbances, stereotyped and repetitive behaviors, social interaction deficits and sensorimotor impairments.

Additionally, we report here a deficit in PC in the vermis (sub-lobule 7cb) of male animals, in line with previous reports in this animal model^16,33^. These findings are in relevance with clinical data that report vermal hypoplasia in ASD patients. The role of the vermal part of the cerebellum is still not clearly determined. Transcranial magnetic stimulation of this cerebellar area in human provoked impaired visual motion discrimination suggesting a role of the cerebellar vermis in visual motion processing^55^. In non-human primate it has been demonstrated that the cerebellar vermis receives projections from motor areas including the primary motor cortex^56^. In relation, we show here a modest but significant decrease of the neuronal number in the M1/M2 motor cortex in poly I:C males. These findings could be indicative of a connectivity dysfunction between the motor cortex and the cerebellum occurring only in male animals.

Vargas et al.^57^, have reported a microglial activation within the cerebellum of ASD patients. This was further corroborated by a later study showing microglial activation mediated by TLR3-poly I:C binding^58^. Given these clinical data, we have examined microglial status in poly I:C animals but found no morphological alterations, in accordance with Hui et al. using the poly I:C mouse model^59^.Together, these results indicate that behavioral deficits displayed by male mice exposed prenatally to poly I:C occur in the absence of protracted neuroinflammation in the cerebellum.

In a previous study, we recently reported that VPA mouse models of ASD also exhibit autism-like behavioral phenotype and less PC^19^. The main differences between the two studies are that VPA treatment affected similarly both males and females, except for the social interaction paradigm that was not alerted in VPA females. Here, poly I:C treated females seemed better protected against MIA as they showed mild and specific behavioral and cellular deficits. These results highlight the heterogeneity of symptoms found in ASD and indicate that males are differentially vulnerable to various environmental insults occurring during pregnancy. The basis for this male bias is unknown with theories including the “extreme male brain”, hormonal differences, and genetic influences (for a review see^60^.

## Conclusion

We report here that a single poly I:C injection on E12.5 negatively affects behavioral neurodevelopmental parameters, exploratory behavior and sociability and that these deficiencies are somewhat more pronounced and of different nature in males than in females. These findings are reminiscent of clinical and epidemiological observations showing higher incidence of ASD in males. This stresses the need to investigate how gender may protect against the effects of MIA in ASD and emphasize the importance of identifying the underlying biological parameters in both sexes in animal models relevant to neurodevelopmental disorders such as ASD.

## Supplementary information

Supplementary Figure 1

Supplementary Table 1

Supplemental legends
