# Supplementary figures and images for: Sex-dependent behavioral deficits and neuropathology in a maternal immune activation model of autism
Source: Transl Psychiatry. 2019 Mar 28;9:124. doi: 10.1038/s41398-019-0457-y (PMC6438965; doi:10.1038/s41398-019-0457-y)

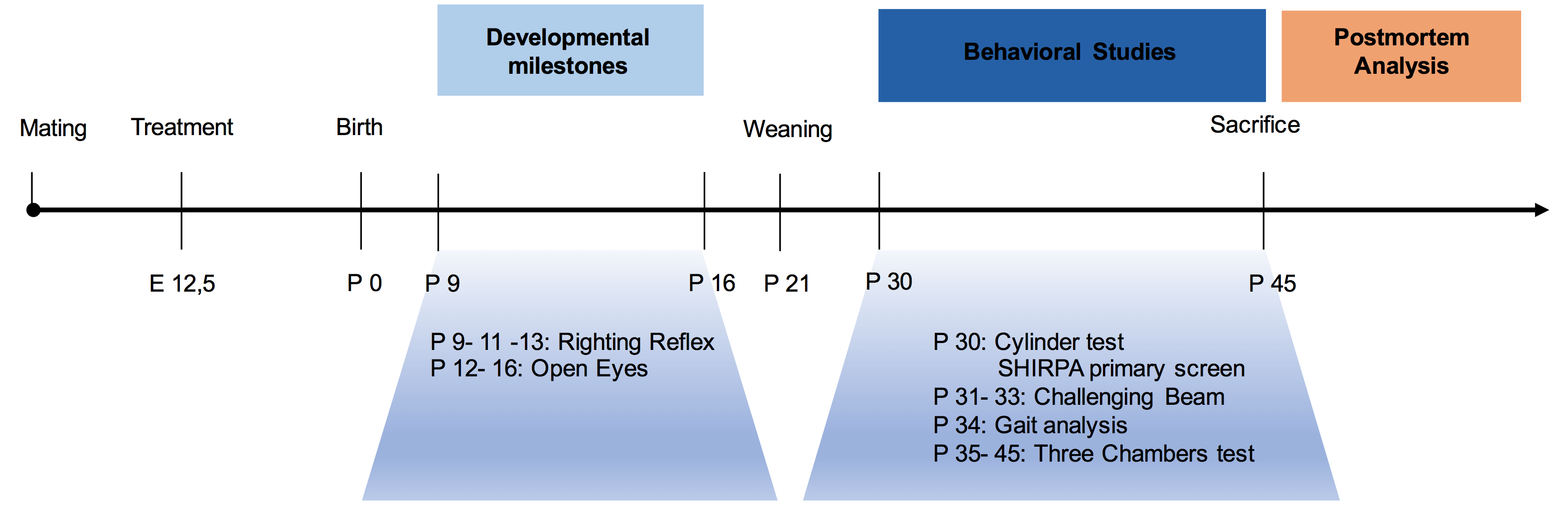

Supplement: Supplementary file 1 — Supplementary Figure 1 [file 41398_2019_457_MOESM1_ESM.tif]
