# Supplementary material for: Sex-dependent behavioral deficits and neuropathology in a maternal immune activation model of autism
Source: Transl Psychiatry. 2019 Mar 28;9:124. doi: 10.1038/s41398-019-0457-y (PMC6438965; doi:10.1038/s41398-019-0457-y)
Supplement: Supplementary file 2 — Supplementary Table 1 [file 41398_2019_457_MOESM2_ESM.pdf]

<u>Supplementary Table 1</u>: Stereological sampling parameters in various brain regions implicated in motor control and coordination

<table>
<tr><th>Brain region</th><th>Neurons counted/staining</th><th>No. of sections</th><th>Counting frame area (μm)</th><th>Sampling grid area (μm)</th></tr>
<tr><td>Sim</td><td rowspan="6">PC/cresyl</td><td>4</td><td rowspan="7">50 x 50</td><td rowspan="4">130 x 170</td></tr>
<tr><td>6cb</td><td>4</td></tr>
<tr><td>Crus I</td><td>5</td></tr>
<tr><td>Crus II</td><td>4</td></tr>
<tr><td>PM</td><td>4</td><td rowspan="2">170 x 130</td></tr>
<tr><td>7cb</td><td>3</td></tr>
<tr><td>SNc</td><td>TH+/ anti-TH mouse</td><td>5</td><td>150 x 150</td></tr>
<tr><td>Striatum</td><td>Total neurons/ anti-NeuN rabbit</td><td>12</td><td>40 x 40</td><td>375 x 325</td></tr>
<tr><td>M1/M2</td><td>Total neurons/ anti-NeuN rabbit</td><td>6</td><td>40 x 40</td><td>375 x 325</td></tr>
</table>
