# Supplementary material for: Sex-dependent behavioral deficits and neuropathology in a maternal immune activation model of autism
Source: Transl Psychiatry. 2019 Mar 28;9:124. doi: 10.1038/s41398-019-0457-y (PMC6438965; doi:10.1038/s41398-019-0457-y)
Supplement: Supplementary file 3 — Supplemental legends [file 41398_2019_457_MOESM3_ESM.docx]

## Supplementary Figure 1: Experiment timeline.

For mating, 3 females were transferred to the male cage and the presence of vaginal plug designated gestational day 0. Pregnant females where treated with either polyinosinic-polycytidylic acid (20 mg/kg) or NaCl 0.9 % on embryonic day 12.5 (E12.5). Sex and age matched pups where separated from the dams on postnatal day 21 (P21) and raised by groups of 4 in a randomized fashion to avoid litter effects. Comprehensive behavioral screening was performed between postnatal days 30 and 45 (P30- 45). Mice where then sacrificed and brains were harvested for histological analysis (P45). For details about groups organization, see the Animals section in Materials and Methods.

## Supplementary Table 1: Stereological sampling parameters in various brain regions implicated in motor control and coordination.

SIM: simple lobule ; PM: paramedian lobule ; SNc: substantia nigra *pars compacta* ; M1/M2: primary and secondary motor cortex ; PC: Purkinje cells ; TH: tyrosine hydroxylase
